# Influence of silver nanoparticles on the tissue reaction of polyacrylic acid-based gel

**DOI:** 10.1590/acb370504

**Published:** 2022-08-12

**Authors:** Jéssica Mariana Bonete, Jacqueline Roberta Tamashiro, Fábio Friol Guedes de Paiva, Geisiany Maria de Queiroz-Fernandes, Éder Guidelli, Oswaldo Baffa, Angela Kinoshita

**Affiliations:** 1 Graduate student. Centro Universitário Sagrado Coração – Center for Health Sciences – Bauru (SP), Brazil.; 2 PhD. Universidade do Oeste Paulista – Postgraduate Program in Environment and Regional Development – Presidente Prudente (SP), Brazil.; 3 PhD. Centro Universitário Sagrado Coração – Center for Health Sciences – Bauru (SP), Brazil.; 4 PhD. Universidade de São Paulo – Faculty of Philosophy, Sciences and Letters of Ribeirão Preto – Ribeirao Preto (SP), Brazil.

**Keywords:** Hydrogels, Nanoparticles, Silver, Antioxidants

## Abstract

**Purpose:**

To study the influence of silver nanoparticles (AgNP) on tissue reaction when incorporated into a polymeric matrix of polyacrylic acid-based (Carbopol^®^) gel as a proposal for a new low-cost type of biomaterial that is simple to manufacture for use as an antimicrobial and antioxidative dressing.

**Methods:**

*In-vivo* tests of implantation in the subcutaneous tissue of the back of rats were performed using polyethylene tubes in three situations: empty, only the gel, and gel incorporated with AgNP. Then, the tissue reaction was studied by counting inflammatory cells. Additionally, *in-vitro* tests of the antioxidative and antimicrobial activity of AgNP were performed. The radical 2,2 diphenyl-1 picrylhydrazyl (DPPH) was used to test the antioxidative activity of AgNP using electron spin resonance. The antimicrobial activity of AgNP was determined by minimum inhibitory concentration against the microorganisms: *Staphylococcus aureus*, *Pseudomonas aeruginosa*, and *Escherichia coli*.

**Results:**

The results indicated that AgNP presents antioxidative activity and was able to inhibit the growth of the microorganisms tested. The addition of AgNP in Carbopol^®^ did not alter the tissue inflammatory response (p*>*0.05, Kruskal-Wallis’s test).

**Conclusions:**

The new biomaterial is promising for future use as a dressing for its beneficial properties for regenerative processes.

## Introduction

Antimicrobial therapies using nanoparticle incorporation to treat infected wounds have been highly applied due to their efficiency. Silver nanoparticles (AgNP) are effective against several microorganisms such as *Staphylococcus aureus*, *Bacillus subtilis*, *Pseudomonas aeruginosa*, *Klebsiella pneumoniae*, and *Escherichia coli* 1. AgNP presents a high surface area due to its very small size and high dispersion. Silver is a safe and effective bactericidal metal, because it is non-toxic to animal cells and highly toxic to bacteria^2^. Almeida *et al*.^3^ found no cytotoxic effect of AgNP delivered by a *Hancornia speciosa* biomembrane, prepared with up to 0.4% of AgNP. Other studies indicate that AgNP toxicity is directly related to the dose and particle size used. The physicochemical properties of AgNPs allowed the development and incorporation of the nanoparticles into new products, for example, for medical use as dressings for wounds. Especially in the case of burns, the best healing process is evident when treated with AgNP^4^. Dressings containing AgNP have higher efficiency in treatments and wound healing^5^, providing a decrease in infection rates, and reduction in antibiotic use and treatment cost^6^.

The development of new types of dressing with biocompatible synthetic polymers in combination with antimicrobial agents has presented an excellent performance in the treatment of wounds and burns^7^. Polyethylene terephthalate meshes were coated with polyacrylic acid anchoring AgNPs. This coating containing carboxylic groups has electrostatic interactions favorable to Ag cations, increasing AgNP carrying capacity^8^. Studies state that hydrogels based on copolymers (methacrylated chondroitin sulfate and acrylic acid) are excellent carriers for a wide variety of therapeutic agents for drug release^9^. Bonete *et al*.^10^ incorporated AgNP to latex of *Hancornia speciosa* to compose a functional biomaterial.

In addition, antimicrobial and antioxidative activity is important in regenerative processes. Free radicals that lead to oxidative stress may cause biological damage, including slowing the healing process. Literature reports some investigations about the antioxidative potential of metal nanoparticles^11^ important in the context of medical application. Thus, the new biomaterial with low-cost comprising AgNP aggregates all these properties, representing a good alternative. However, the study of the tissue reaction is crucial for its future application.

The present study describes the development of a wound dressing consisting of polyacrylic acid-based gel with AgNPs, a study of the tissue reaction after the subcutaneous implant, and an evaluation of its antimicrobial and antioxidative properties for future use as a low-cost dressing.

## Methods

### Silver nanoparticles synthesis and gel preparation

AgNP were obtained by chemical reduction of silver nitrate solution 16 mmol·L^-1^ (AgNO_3_) by sodium borohydride solution 32 mmol·L^-1^ (NaBH_4_). Ultrapure miliQ water was used as a solvent for both. The solutions were mixed and homogenized on a magnetic stirrer at 1,800 rpm and room temperature. The system was stirred for 1 h to ensure a complete reduction of the silver. The concentration of colloidal suspension was 431 μg·mL^-1^ of Ag. AgNP formation is characterized by a yellowish color of the solution, with the plasmon peak in the ultraviolet-visible (UV-Vis) spectrum around 400 nm^12^. Transmission electron microscopy (TEM) images of AgNP were acquired using a JEOL-JEM-100 CXII instrument. The gel was obtained with 1 mL of AgNP (0.043% or 431 μg·mL^-1^) solution added to 65 mg (6.5%) of polyacrylic acid/Carbopol 940® (carbomer-940), a water-soluble acrylic polymer that increases the viscosity of the solution until a gel is obtained, which we will call CPAg. For the tissue reaction tests, a gel without the addition of nanoparticles was formulated (CP) using the same proportion (6.5%).

### Antimicrobial activity of AgNPs

The minimum inhibitory concentration (MIC) of AgNPs was determined against *E. coli* (ATCC 25922, clinical isolate), *S. aureus* (ATCC 25923, clinical isolate), and *P. aeruginosa* (ATCC 25619), from American Type Culture Collection. These samples are kept in the collection in the Laboratory of the Centro Universitário Sagrado Coração, Bauru, SP, Brazil. Bacteria were grown on Mueller-Hinton agar at 37 °C for 24 h, followed by dilution in 0.9% saline to reach a final concentration of 2.5 × 10^5^ CFU·mL^-1^, according to the standard protocol developed by Clinical & Laboratory Standards Institute^13^. After serial dilution of the AgNP solution (431 to 0.21 μg·mL^-1^) and the positive ampicillin control (25 to 0.006 μg·mL^-1^) in a 96-well microplate (Kasvi®), 100 μL of adjusted bacterial inoculum (2.5 × 10^5^ CFU) was added to each well. The plates were incubated at 37 °C for 24 h. For bacterial growth analysis, 20 μL of sterile 0.01% resazurin solution was added to each well. The plates were incubated for 1 h at 35 °C in a bacteriological oven for further visual reading. In this revealing system, the presence of the blue color represents the absence of viable cells, while the red color is interpreted as the presence of viable cells. The tests were performed in triplicate.

### Antioxidative properties

Electron spin resonance (ESR) was used to determine the anti-free-radical activity of the synthesized AgNP. A volume of 200 μL of AgNP solution was mixed with 200 μL of the solution containing 2 mM free radical 2,2 diphenyl-1 picrylhydrazyl (DPPH). Methanol was used as a reference. After each reaction, the resulting solution was transferred to quartz tube to record the spectrum. ESR spectra were acquired sequentially every 3 min in the ESR spectrometer JEOL FA 200 X Band, at room temperature, to study the kinetics reaction between the nanoparticles and the radical. The spectrometer parameters for the acquisition of DPPH spectra were: central field 345 mT, scan width 10 mT, scan time 1 min, amplitude modulation 0.1 mT, microwave power 1 mW, modulation frequency 100 kHz, and microwave frequency 9.5 GHz.

### Tissue reaction in-vivo experiment

**Figure 1:**
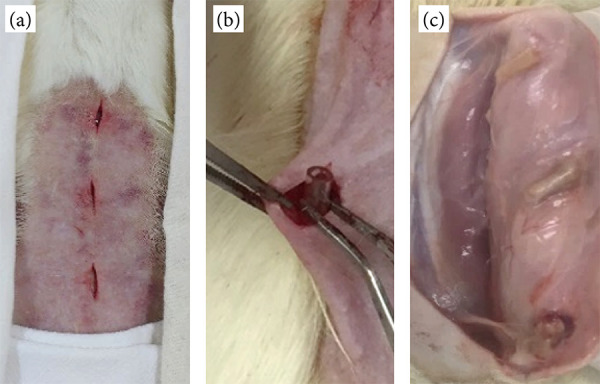
Incisions made in the dorsal region of the rat to implant biomaterial. **(b)** Insertion of biomaterial guided by a polyethylene tube. **(c)** After euthanasia, the access to the implanted biomaterials.

This study was approved by the Committee of Ethics in Animal Use of Centro Universitário Sagrado Coração, protocol number 7375290415. Eighteen male adult *Rattus norvegicus albinus*, Wistar lineage, were used. Initially, deep sedation was induced by intraperitoneal application of muscle relaxant xylazine hydrochloride (10 mg·kg^-1^) (Rompun, Bayer, São Paulo, SP, Brazil) and ketamine hydrochloride (90 mg·kg^-1^) (Dopalen, Ceva, São Paulo, SP, Brazil). Then, the epilation process of the dorsal region and antisepsis with chlorhexidine (2%) were performed. In each animal, three incisions of 5 mm were made in the skin, followed by dilatation of tissues (Fig. 1a). The biomaterials were implanted into the subcutaneous tissue. For the implant of biomaterial, a sterile polyethylene tube of 1 cm in length was used, filled as follows: with gel associated with AgNPs (CPAg), pure gel (CP), and an empty tube (Fig. 1b). After implantation, the tissue was sutured with a 4 silk suture. After the surgery, as an analgesic procedure, 25 mg·kg^-1^ of monohydrated dipyrone (D-500, Zoetis, Campinas, SP, Brazil) was administered subcutaneously twice daily for three days. After the observation periods of seven, 15, and 60 days, six animals were submitted to euthanasia by overdose administration of barbiturates sodic thiopental 150 mg·kg^-1^ (Thiopentax; Cristália, Itapira, SP, Brazil) associated with 10 mg·mL^-1^ lidocaine chlorydrate (Lidovet; Bravet, Rio de Janeiro, RJ, Brazil) by intraperitoneal administration. The area containing tissues around the implanted material was collected to perform the microscopic analysis (Fig. 1c).

For microscopic analysis, the samples were fixed in 10% formaldehyde for 48 h, and conventional procedures for histological preparation were followed. The histological sections were obtained in the longitudinal direction with 6-?m thickness and stained with hematoxylin and eosin (HE). Eight images of each histological section were taken in a standardized way with Image-Pro® Plus software (400x magnification), microscope optic Nikon Eclipse 80i. Subsequently, quantification and identification of the types of mononuclear cells, polymorphonuclear leukocytes, and giant cells present in the images were performed using ImageJ software^14^. Each type of cell was recorded over periods of seven, 15, and 60 days. The nonparametric Kruskal-Wallis’ test and Student-Newman-Keuls were used for comparison, and the samples were considered statistically different when p<0.05.

## Results

### Morphology of the silver nanoparticles

**Figure 2:**
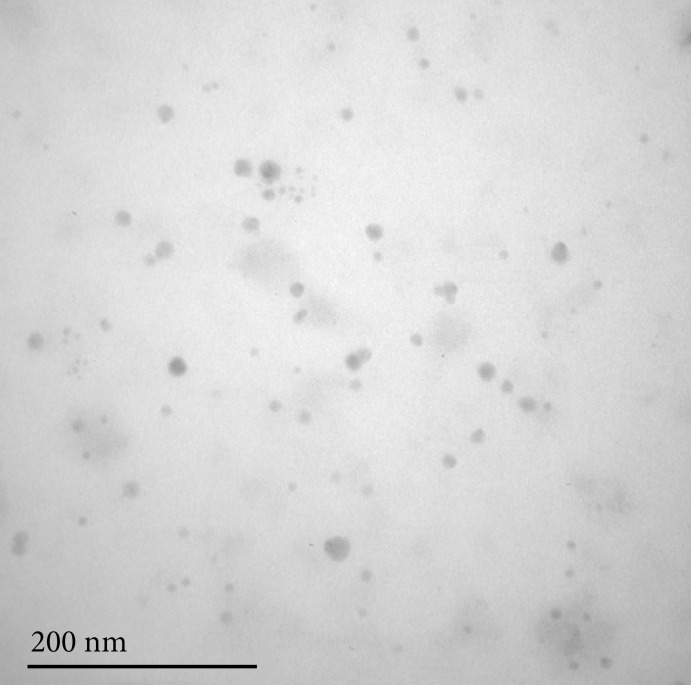
Transmission electron microscopy image of silver nanoparticlessolution. The nanoparticles present diameters of 10 to 15 nm.

Figure 2 shows the TEM micrograph of AgNPs. As can be observed, nanoparticles are spherical and present particles with size between 10 to 15 nm.

### Antimicrobial activity

AgNPs MIC was 26.96 μg·mL^-1^ for *P. aeruginosa* and 13.48 μg·mL^-1^ for *S. aureus* and *E. coli*. Ampicillin was used as a positive control and showed no efficient inhibition against *P. aeruginosa*, and its MIC was 25 μg·mL^-1^ for *E. coli*. and 0.2 μg·mL^-1^ for *S. aureus.*

Antioxidative activity

**Figure 3:**
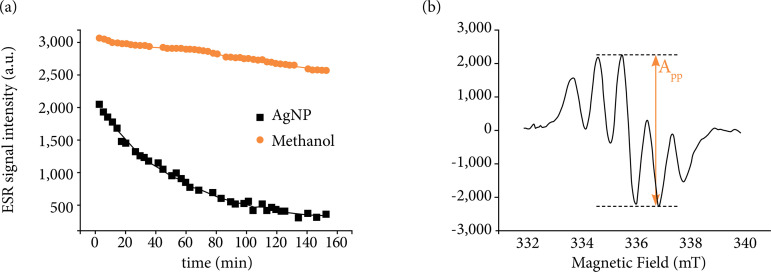
**(a)** ESR spectrum of DPPH showing the intensity of the central line. **(b)** ESR signal intensity of DPPH overtime after the reaction with AgNP and methanol.

ESR: Electron spin resonance; DPPH: 2,2 diphenyl-1 picrylhydrazyl; AgNP: silver nanoparticles.

ESR spectroscopy is used to detect unpaired electrons, present in free radicals, for example. The DPPH is a compound that contains stable free radical molecules and it is, therefore, detectable by ESR^15^. Its spectrum is shown in Fig. 3a; when reacting with an antioxidant substance, the amplitude of the ESR spectrum is reduced. Figure 3b shows the amplitude of the center line of the DPPH spectrum as a function of reaction time with the AgNP solution (black line) and with Methanol (red line). Data were fitted with the Eq. 1:


$$I=I_{0}+Ae^{-t/T}$$ (1)

In which:

*I*: the signal intensity at time *t*;
*I*_0_: the initial signal intensity;
*A*: a constant;
*T*: a characteristic time constant.

The time constant T for AgNP, related to the antioxidative activity, is 52±2min, while for methanol it is T = 227±40 min.

### Microscopic analysis

**Figure 4:**
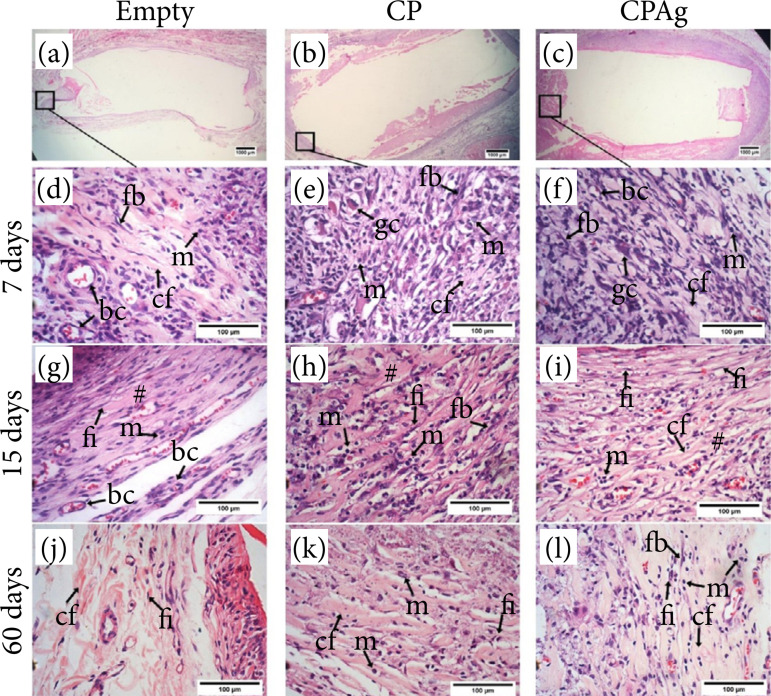
Photomicrographs show the subcutaneous tissue of the implantation of the materials. The region of the tissue in which the microscopic analyses were performed are demarcated with a square for **(a)** the empty tube, **(b)** CP, and **(c)** CPAg, (d, g, j) represent photomicrographs from the period of 7 days after surgery, (e, h, k) 15 days and (f, i, l) 60 days. At seven days, the tissue reaction evidences the presence of intense inflammatory infiltrate and a dense deposition of collagen fibers (

Figure 4 shows photomicrographs of the empty polyethylene tube (Fig. 4a), containing pure carbopol gel (Fig. 4b) and carbopol gel incorporated by nanoparticles (Fig. 4c) of tissue after implantation. Collagen fibers and inflammatory infiltrate surrounded the opening of the empty polyethylene tube (E) (Fig. 4a). The same features were observed for the ones that were filled with the carbopol gel (CP) and with the carbopol gel incorporated silver nanoparticles (CPAg) (Figs. 4b and 4c, respectively).

At seven days after the implant, the presence of remodeling connective tissue was evident in both groups of materials; it was possible to observe a dense deposition of collagen fibers (Figs. 4d-4f). An intense mononuclear inflammatory infiltrate was seen, and the presence of some giant cells was registered, as shown in Fig. 4e.

At 15 days after the implant of the materials, the presence of dense fibrous tissue was observed in E (Fig. 4g), showing blood capillaries and small infiltration of mononuclear cells. The tissue exposed to CP (Fig. 4h) and CPAg (Fig. 4i) 15 days post-surgery also formed a dense fibrous tissue, but it presented an infiltration of cells of the most intense inflammatory infiltrate. After 15 days post-surgery, the presence of a chronic inflammatory infiltrate was more intense.

At 60 days after implantation, the region at the opening of the empty tube (E) contained a fibrous capsule, remodeling connective tissue with collagen fibers arranged parallel to each other in contact with the material; the presence of fibrocytes, adipocytes, and resident cells was observed (Fig. 4j). Figures 4k and 4l show the formation of dense connective tissue with the presence of fibroblasts and fibrocytes, an extracellular matrix with good structure, with collagen fibers arranged parallel and mild mononuclear inflammatory infiltrate in both groups. They show a very weak inflammation process, almost nonexistent in the regions in which CP and CPAg were implanted.

Histomorphometry analysis

**Figure 5:**
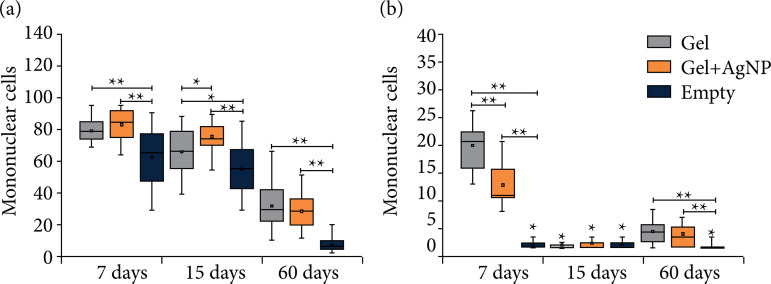
Box-plot graphs showing **(a)** mononuclear cells and **(b)** polymorphonuclear cells quantified in the photomicrographs (eight images were made at 40x magnification, for each slide), at periods of seven, 15, and 60 days.

The mononuclear cells (Fig. 5a) and polymorphonuclear cells (Fig. 5b) quantified in the photomicrographs (eight regions by image, 400x magnification) were represented in box-plot graphs. The number of polymorphonuclear cells for CP was relatively superior to CPAg and the empty tube at seven days after the surgery (p < 0.0001, Kruskal-Wallis’ test). After 15 days, the amount was similar between the three groups. After 60 days, CP and CPAg (p=0.1499, Kruskal-Wallis’ test) showed larger amounts of this cell type compared to the empty tube E (Fig. 5b) (p < 0.0001, Kruskal-Wallis’ test), except for at 15 days (p=0.4309, Kruskal-Wallis’ test).

AgNP: silver nanoparticles; *p<0.05; **p<0.0001.

## Discussion

Silver nitrate (AgNO_3_) was used as a source of silver ions in chemical reduction with NaBH_4_, and the solution turned to a yellowish brown color. The nanoparticles obtained in this synthesis presented diameters of 10 to 15 nm, similar to that reported in the literature. Cheon *et al*.^16^ synthesized AgNP with spherical, triangular, and disk shapes. They demonstrated the dependency of the shape in the antimicrobial activity because the surface area modifies the Ag release. The spherical shape, the same used in this work, was more effective against *S. aureus* and *P. aeruginosa*.

In the MIC experiment, the AgNP performed better than Ampicillin against *P. aeruginosa* and *E. coli*. Bhowmick and Koul^17^ and Bhowmick *et al*.^18^ found MICs of AgNPs similar to this work. These microorganisms are very common in bacterial biofilm in chronic wounds, causing a prolonged inflammatory phase and slowing or preventing the healing process^19^.

The potential of the silver nanoparticles was also evidenced by other authors, and AgNPs dispersed in polymer matrix showed high efficacy against *E. coli*, *S. aureus*, and *P. aeruginosa* 20 ^,^ 21. Cencetti *et al*.^22^ described very significant antimicrobial activity against the gram-positive *S. aureus* and gram-negative *P. aeruginosa*, results similar to those found in this work. In addition, bacteria are more susceptible to silver toxicity because of their low structural and functional level and relatively small cell size. Recently, Flauzino Júnior *et al*.^23^ reported the antimicrobial activity against *S. aureus* and *E. coli* of a compound formed by chitosan, poy (lactic acid), and AgNP and its cytocompatibility, in tests with fibroblast cells. For genotoxic effects, high concentrations of AgNP are required^3^.

Literature reports AgNP do not exhibit toxicity at concentrations of 8 to 128 μg·mL^-1^ 24. The concentration of CP incorporated in the tested AgNP is 4,315 ppm. Considering the density of water at 1 g·mL^-1^, the gel concentration used in this work was higher than the mentioned value, although it did not cause any difference in the inflammatory response. But the MIC experiment showed that 26.96 μg·mL^-1^ is sufficient to inhibit the important microorganisms present in chronic wounds. Using microdilution tests, Panáček *et al*.^25^ demonstrated that only 1 μg·mL^-1^ of AgNP is sufficient to potentiate the antimicrobial effect of several antibiotics, against bacteria, even those considered very resistant. The selection of bacteria resistant to antibiotics caused the need to incorporate bactericidal agents to potentiate the action of drugs. In addition, it is proven that excessive inflammation impairs and delays the tissue repair process. The topical application of agents with antimicrobial and antioxidative properties contributes to the process of tissue repair and return to normal tissue conditions. The effectiveness of nanoparticles against several pathological microorganisms justifies the use of AgNP as one of the main antimicrobial agents used in dressings to aid in the wound healing process^22^. The efficiency of nanoparticles against several microorganisms justifies their great use as dressing material for wound healing^26^.

Antioxidative activity is usually assessed by spectrophotometry^27^, which monitors this activity by UV-Vis spectrophotometry. In this case, due to the staining of the AgNP solution (yellow-brown), the use of ESR represents an advantage, in addition to directly evaluating the annihilation of the radical. Also, the antioxidative activity is an important feature of dressings as free radicals delay healing processes.

Concerning the tissue reaction, the pattern of tissue repair that includes cell migration and formation of inflammatory infiltrate, formation of new blood capillaries, and proliferation of fibroblasts that are responsible for the deposition of an extracellular matrix^28^ was observed for all tested materials. At seven days post-surgery, an acute inflammatory infiltrate predominated, with the presence of polymorphonuclear cells, giant cells, and intense neovascularization. Over time, there was a greater deposition of extracellular matrix evidenced by a mass of collagen fibers. After 15 days post-surgery the presence of a chronic inflammatory infiltrate was more intense.

At 60 days, the connective tissue was well structured; for the empty polyethylene tube loose connective tissue, fibroblasts, with the presence of adipose tissue, and a dense connective tissue were formed. For CP and CPAg, some mononuclear cells were still visible in this period. Silva *et al*.^29^ also observed an inflammatory reaction of Cabopol® and, when associated with another drug with anti-inflammatory potential, pure carbopol hydrogel showed a greater inflammatory response. However, nothing that could harm tissue regeneration since this hydrogel is considered an excellent topical delivery system, with high viscosity, good biocompatibility, bioadhesive properties, and good thermal stability. In addition, it favors moisture balance, which is essential for the healing process^29^.

In the images, the process of angiogenesis and formation of frightening tissue that characterizes the granulation tissue, which is essential for the repair process^30^, is also observed. The polyethylene tube used in the implantation of the biomaterials facilitated the localization of the tested material in the subcutaneous tissue. Polyethylene is a non-absorbable material, but it does not significantly interfere with the inflammatory response process, as demonstrated by observing the 60-day photomicrographs (Fig. 5j). The histopathological analysis of the tissue reaction to the polyethylene tube reveals the formation of granulation tissue, inflammatory infiltrate, and a fibrous capsule, regressing over time and disappearing after approximately 12 weeks^31^.

Despite the similar tissue reaction among the three materials, the empty tube implantation showed a smaller number of mononuclear cells compared to CP and CPAg (Fig. 5a) in all periods analyzed (p < 0.0001). The tissue reaction observed presented a regression in the inflammatory infiltrate over time for both groups.

The quantified mononuclear cells were similar for all periods for CP and CPAg, with no statistically significant difference (p>0.05, Kruskal-Wallis’ test). Thus, we observed that the addition of AgNP did not alter the tissue response when compared to CP, and the tissue reaction was physiologically similar between the three materials. Therefore, the present work demonstrated the possibility of using a commercial hydrogel already known to release AgNP and thus having an antimicrobial, antioxidant, safe, low-cost dressing that will prevent infections during healing processes.

## Conclusions

The results indicated that the proposed biomaterial presents a favorable tissue reaction and it is physiologically like the Carbopol 940® polymer gel already used in the pharmaceutical industry in the development of gels and ointments. The antimicrobial activity and the antioxidant action presented by AgNP are important properties for the tissue repair process, favoring it in cases of non-healing wounds. The results indicate a high potential of this biomaterial for future application as dressings.
